# ‘Tweaking’ the model for understanding and preventing maternal and neonatal morbidity and mortality in Low Income Countries: “inserting new ideas into a timeless wine skin”

**DOI:** 10.1186/s12884-016-0803-5

**Published:** 2016-01-25

**Authors:** Michael K. Mwaniki, Evaline J. Baya, Faith Mwangi-Powell, Peter Sidebotham

**Affiliations:** University Research Co., LLC (URC) 7200 Wisconsin Avenue, Ste. 600, Bethesda, MD 20814 USA; Afya Research Africa, P.O. Box 20880, 00202 Nairobi, Kenya; Nairobi University, College of Health sciences, Kenyatta National Hospital, P. O. Box 19676-00202, Nairobi, Kenya; Mental Health & Wellbeing, University of Warwick, Medical school Building, Gibbet Hill Campus, Coventry, CV4 7AL UK

**Keywords:** Five pronged framework, Three delay Model, Maternal, Neonatal, Morbidity, Mortality, Low Income Countries

## Abstract

**Background:**

Maternal and neonatal morbidity and mortality in Low Income Countries, especially in sub-Saharan Africa involves numerous interrelated causes. The three-delay model/framework was advanced to better understand the causes and associated

Contextual factors. It continues to inform many aspects of programming and research on combating maternal and child morbidity and mortality in the said countries. Although this model addresses some of the core areas that can be targeted to drastically reduce maternal and neonatal morbidity and mortality, it potentially omits other critical facets especially around primary prevention, and pre- and post-hospitalization continuum of care.

**Discussion:**

The final causes of Maternal and Neonatal mortality and morbidity maybe limited to a few themes largely centering on infections, preterm births, and pregnancy and childbirth related complications. However, to effectively tackle these causes of morbidity and mortality, a broad based approach is required. Some of the core issues that need to be addressed include:-i) prevention of vertically transmitted infections, intra-partum related adverse events and broad primary prevention strategies, ii) overall health care seeking behavior and delays therein, iii) quality of care at point of service delivery, and iv) post-insult treatment follow up and rehabilitation. In this article we propose a five-pronged framework that takes all the above into consideration. This frameworks further builds on the three-delay model and offers a more comprehensive approach to understanding and preventing maternal and neonatal morbidity and mortality in Low Income Countries

**Conclusion:**

In shaping the post 2015 agenda, the scope of engagement in maternal and newborn health need to be widened if further gains are to be realized and sustained. Our proposed five pronged approach incorporates the need for continued investment in tackling the recognized three delays, but broadens this to also address earlier aspects of primary prevention, and the need for tertiary prevention through ongoing follow up and rehabilitation. It takes into perspective the spectrum of new evidence and how it can be used to deepen overall understanding of prevention strategies for maternal and neonatal morbidity and mortality in LICS.

## Background

In the last decade concerted efforts in low income countries (LICS) have led to a decline in the overall mortality of children under the age of five years [1]. Sustaining immunization programs and even introducing new vaccines for invasive bacterial diseases (haemophilus and pneumococcal infections), and combating the burden of falciparum malaria are some of the notable strategies that have received consistent global funding and attention especially in sub-Saharan Africa [2, 3]. It is however worth noting that most of the considerable decline in morbidity and mortality has occurred in children, post the neonatal and young infant age group (>2 months of age) [2, 3]. Comparatively, neonatal mortality has shown modest reduction [4, 5]. Furthermore, evidence shows that neonatal deaths as proportion of total under-five mortality has risen over the same time [6]. This may suggest that our generic approach to combating under-five mortality has not invested and deployed strategies that specifically address the underlying causes of neonatal morbidity and mortality in LICS.

Improving maternal health is a key global agenda [7]. Although some progress has been noted, the goal of reducing maternal mortality ratio (MMR) by three quarters by 2015 remains unmet by most of the LICS [8]. In most of the sub Saharan African region, MMR remains in the very high category (510/100,000 live births), more than double the global average of 210/100,000 [9]. Moreover, the lifetime risk of maternal death in the LICS especially sub Saharan Africa remains as high as 1 in 160 compared to 1 in 3700 for developed countries [8]. In a nutshell, Maternal and Neonatal morbidity and mortality significantly contribute to the overall global morbidity and mortality. Most of this morbidity and mortality will continue to occur in LICS such as the sub-Saharan African region. It is therefore clear that with the dawn of the post 2015 era, the question ‘where is the M in MCH?’ continues to be highly valid thirty years after it was first asked [10]. However, the more ‘wholesome’ question to consistently ask in every programmatic or research investment to combat maternal and child mortality beyond 2015 may be where is the ‘M & N’ in MNCH?

Maternal and neonatal morbidity and mortality in LICS especially in sub-Saharan Africa involve numerous interrelated causes [11]. The three-delay model/framework was advanced to better understand the causes and associated contextual factors [12]. The model comprises delay in deciding to seek care (delay 1), delay in reaching the health facility (delay 2), and delay in receiving quality care once at the health facility (delay 3) [12]. Since its advancement two decades ago, the three delay model/framework has remained relatively unchanged [12]. It continues to inform many aspects of programming and research on combating maternal and child morbidity and mortality in LICS. Although this model addresses some of the main areas that can be targeted to drastically reduce maternal and neonatal morbidity and mortality, it potentially omits other critical facets especially around primary prevention, and pre- and post-hospitalization continuum of care.

The post 2015 global deliberation needs to concisely articulate where to invest in order to rapidly accelerate the gains made in a push towards achieving the millennium development goals (MDGs), particularly 4 & 5. In doing so, several issues need to be taken into consideration. First, LICS have witnessed reasonable economic growth during the last two decades with many poised to acquire middle or ‘near’ middle income status [13]. This may result in meaningful national and regional funding being available for maternal and child health programs to complement global investments. Second, many of these countries have also witnessed rapid urbanization which may also require a rethink in public health approaches [13]. Third, the unprecedented knowledge from more than two decades of public health programming and research centered mainly on the three delay model now exists. Finally, the epidemiological mix of the major causes of maternal and newborn mortality may have changed since the three-delay model was advanced [14–17]. For example, HIV and AIDS may now be responsible for a sizeable proportion of maternal morbidity and mortality in some countries [9]. Similarly, Group B streptococcus contribution to neonatal sepsis in LICS was still largely undocumented two decades ago but now may be responsible for a fair proportion of sepsis-related neonatal deaths in LICS [14–17]. Given all the above, there is need to update the three-delay model to accurately shape the post 2015 global health agenda . In this article we draw on our own research in LICS and an understanding of the wider research literature to propose a more comprehensive five-pronged framework (Table 1). This framework, building on the three-delay model, is discussed below.

**Table 1:** Five-pronged framework for understanding and preventing Maternal-Neonatal morbidity and mortality in low income countries

Foetal-Maternal strategies	Original component of the three-delay model advanced in 1994			Post-discharge follow up and rehabilitation
(Primary Prevention)	(Secondary Prevention)			(Tertiary Prevention)
Prong 1	Prong 2	Prong 3	Prong 4	Prong 5
• Prevention of vertical transmission of infections • Prediction and Preventing intra-partum related neonatal morbidity and mortality • Vaccines against causes of early neonatal sepsis especially Group B Streptococcus and other diseases including HIV|AIDS • Chemoprophylaxis against GBS • Effective prevention of mother to child transmission of congenital infections including HIV|AIDS • Barrier preventions e.g provisions of LLITNS^a^ • Family planning, education and economic empowerment of women • Specific focus on adolescent girls	❖ *Addressing delays in decision to seek care* • Education on danger signs during pregnancy and early neonatal life • Community engagement through behavioral change and communication strategies • Demand of care creation	❖ *Addressing delays in reaching point of care once decision is made* • financial incentives e.g. transport refunds • facility output-based financing approaches • referral systems that can help minimize delays that contribute to HIE^b^ • use of community cadres	❖ *Addressing delays in provision of quality services at point of care* • point of care quality improvement approaches • continuous investment in in-service skills development • operational research on how to best offer care at point of service delivery • More research on the role of facility improvement teams • Continuous direct investments in health systems building blocks • Exploring adjunct treatment options for infections and HIE	❖ *Post-discharge follow up and rehabilitation* • expanding of human resource for health cadres with rehabilitation skills (occupational, physiotherapy,& psyscho-social support) in LICS • surveillance systems for post-discharge complications and or sequelea • specific follow up and treatment of obstetrics complications including obstetric fistulas • Ongoing support where long-term impairments are anticipated

^a^LLITN-Long Lasting Insecticide treated mosquito nets

^b^HIE-Hypoxic ischemic Encephalopathy

## Discussion

The final causes of Maternal and Neonatal mortality and morbidity maybe limited to a few themes largely centering on infections, preterm births, and pregnancy and childbirth related complications. However, to effectively address the same, a broad based approach is required. Some of the core issues that need to be addressed include:-i) prevention of vertically transmitted infections, intra-partum related adverse events and broad primary prevention strategies, ii) overall health care seeking behavior and delays therein, iii) quality of care at point of service delivery, and iv) post insult follow up and rehabilitation.

We explore each of this facets below as the core components of our proposed five pronged approach.

### Prong 1: Foeto-Maternal strategies (including prevention of vertical transmission of infections & intra-partum related morbidity and mortality) coupled with broad primary prevention strategies

The foeto-maternal interface represents a common route of transmission of several infections including HIV and AIDS, bacterial and even parasitic infections including congenital malaria [9, 14, 15]. As already described, diseases and conditions around the foeto-maternal interface are now recognized as some of the main causes of maternal and neonatal morbidity and mortality. Of note, Group B Streptococcus (GBS) continue to gain prominence as a major cause of especially early onset neonatal sepsis in LICS [14–19]. Therefore, strategies aimed at addressing this first prong, such as chemo-prophylaxis against bacterial and viral infections e.g Group B *Streptococcus*, HIV and AIDS, and barrier protections through long lasting insecticide treated mosquito nets (LLITNs), offer an opportunity to reduce the burden of maternal and neonatal morbidity and mortality in LICS that need to be continuously supported in LICS. Importantly, approaches to specifically screen for and minimize risk of GBS colonization among pregnant women have shown promising results in reducing early onset sepsis in high income countries [20–22]. Given the grown evidence that GBS is equally significant in LICS there is a need to explore how similar strategies can be rolled out in low income regions. However, it is clear that In spite of the opportunities that portend from the above outlined strategies, operational challenges may hinder their largescale application in LICS. Given this, there is need to continue research investments for viable vaccine options for conditions such as early neonatal sepsis due to group B Streptococci and HIV|AIDS [15].

Apart from neonatal sepsis and vertical transmissions of infections, intra-partum related neonatal morbidity and mortality is a major contributor to neonatal morbidity and mortality [4, 6, 23, 24]. Furthermore, intra-partum related morbidity and mortality may be increasing while that from sepsis is on the decline [6]. Available literature suggests that interventions against intra-partum related neonatal morbidity and mortality in LICS are largely limited to promoting early access (prong 2 & 3) as advocated for in the three delay model, with hardly any direct primary prevention strategies (prong 1), and limited institutionalization of prongs 4 & 5 in respective health care systems as outlined in our proposed framework. Largely, viable evidence based direct primary prevention (prong 1) interventions for perinatal asphyxia including accurate prediction are yet to be fully developed [25–27]. Given this, emphasis must continue to be put into facilitating early access and improving quality of care during labor and delivery. However, there is need to start exploring modalities of rolling out new treatment options that have been shown to reduce long-term neuro sequelea such as therapeutic hypothermia in LICS [28, 29].

It is worth noting that for greater success to be achieved, prong one interventions should also include other broad strategies. Critical areas worth focusing on include family planning, economic and education empowerment of women and girls, and a sustained strategy to address the needs of adolescent girls [30, 31]. By preventing unwanted pregnancies, and offering women opportunities to plan their reproductive choices, evidence suggests that family planning is an effective and low cost approach that may synergistically impact positively on the other available repertoire of strategies to prevent maternal and newborn morbidity and mortality [32]. Recent estimates indicate that family planning may indirectly prevent up to a third of all maternal deaths and an even higher number of neonatal and early childhood deaths [31, 32]. Therefore rapidly scaling up access to, and expanding family planning choices in sub Saharan Africa and other LICS where the current coverage is under fifty percent, should be a core priority in every global, regional and country specific strategic plan that aims to reduce maternal and neonatal deaths [30].

There are nearly 1.5 billion youths in the world today [33]. More than half of them are girls and young women with over 600 million adolescent girls living in low and middle income countries (LMIC) of south east Asia and sub-Saharan Africa [33]. These girls face many challenges ranging from early marriages, lack of general education including sex education, female genital mutilation, gender-based-violence, and poor access to reproductive health services especially family planning services [33]. It is estimated that over 10 % of girls in regions such as the Sub Saharan Africa may have their first child before the age of 16 years [34]. Overall, approximately 16 million births and up to three million unsafe abortions occur among adolescent girls [33, 34]. It is therefore not surprising that pregnancy complications are among the leading causes of death among adolescent girls. Furthermore, still births and newborn deaths may be much higher among adolescent mothers [35]. It is notable that over the last decade, MDGs barely focused on the plight of the adolescent girl. Given the pivotal role of addressing issues affecting adolescent girls as a strategy to improve overall maternal and newborn health, the post 2015 global agenda should invest heavily in this issue as part of the central global MNCH agenda.

In summary, the three-delay model did not encompass the importance of the foeto-maternal interface and its role in combating neonatal morbidity and mortality. In its first ‘delay’, it largely fast-forwards to addressing individual/family/societal/cultural elements [12]. It is however evident that considerable gains in reducing maternal and neonatal morbidity and mortality in LICS may be accrued from consistent investments in prong 1 as articulated in our framework. Investments in prong 1 need to especially bridge funding gaps in research on vertical transmission of infections and intra-partum related morbidity and mortality. In addition, investments in prong 1 should also focus on broad multi-systems and multi-dimensional elements that can hardly be pinned to individual choice/decision to-seek or not-to-seek healthcare services that have traditionally been the focus of the first delay in the three delay model.

### Prong 2 and 3: Addressing delays in decision to seek care and delays in reaching point of care once decision is made (Health promotion during pregnancy)

Prongs two and three of our framework solidifies the continued relevance of all the core aspects of the three-delay model even in the post 2015 global health agenda. Since the development of the three-delay model two decades ago, concerted efforts have been made to understand and address the first two components i.e. delay in making a decision to seek care and delay in reaching a health facility [36]. Ministries of Health in LICS, with considerable support from bilateral funded health programs, have put substantial resources in campaigns to enlighten their populace on most topical issues that centre on eliminating the first delay. These include encouraging focused antenatal care, teaching pregnant women on danger signs during pregnancy and the immediate puerperium including signs of ill health among newborns, and encouraging health facility delivery [36]. Further strategies including establishing more health facilities, having waiting homes near health facilities for pregnant mothers, increasing the number of health workers, and cash incentives to both the pregnant women and health facilities continue to be rolled out in LICS in a bid to further minimize the first and second delays [37]. These strategies seem to have been justified given that most deaths occurred, and continue to occur, in remote rural homes [38]. Importantly, these efforts may be bearing fruits [2, 5, 6].

Further research is needed, especially on innovative approaches that create demand for health services and encourage the use of community cadres for rapid assessment and referral of pregnant women and newborn babies requiring medical attention. Overall, global and in-country investments in LICS towards eliminating these first two central components of the three-delay model need to continue beyond 2015 so that the gains made over the last two decades can be sustained.

### Prong 4: Addressing delays in provision of quality services at the point of care

The third delay represents the most complex aspect of the health care system i.e. the interaction between the healthcare provider and the client. For quality care to occur several requirements must be fulfilled during each interaction. First, a healthcare worker needs to be available. Second, the healthcare worker needs to have the necessary knowledge and skills to diagnose and treat the presenting illness. Third, he or she requires appropriate resources at hand to attend to the situation. Fourth, the healthcare worker need to have put in place an efficient facility ‘Micro-systems’ that ensure each client receives the right care, consistently. Finally, they need to possess the right behavioral attributes to offer the said care in an empathetic and culturally appropriate manner. It is also important to note that the clients’ perceptions of ‘poor’ quality of care may not reflect the true quality of care at the point of service. Nevertheless, these are important perceptions and need to be addressed.

Quality of care however cannot be comprehensively addressed from only a narrow focus on individual healthcare workers and their interaction with clients, but must go deeper to consider the systems issues underlying those interactions. From the onset, improving quality of care in LICS requires broad-based investments in, and monitoring of recognized building blocks for health, and overall health systems strengthening [39]. Global healthcare funding in the last several decades, though laudable, remains predominantly biased towards disease-specific responses rather than health systems responses [40]. Although disease-specific investments may in some instances lead to rapid initial results, institutionalization and sustainability becomes problematic in a weak health system milieu [40]. How to reallocate some existing global as well as in-country funding in LICS to support both disease-specific responses as well as broad health systems responses will be key in driving sustainable improvement in the sector. For starters, it can address some of the quality parameters articulated in the three-delay model including provision of essential supplies, equipping facilities, and staffing among others [12]. However, given that ‘systems’ responses may take longer before results can be realized, it is likely that many funding organizations may be tempted to ‘shy’ away from health systems investments unless there is a sustained global agenda on strengthening health systems in LICS.

Many a times it is easy to perceive quality of care from the availability or non-availability of required inputs (staff, supplies, equipment’s, among others). This is clearly a health system issue as described above. However, another core aspect of quality of care is what happens at care micro-systems level [41]. Even in well-resourced nations with adequately financed health systems, and where equipment, supplies and staffing are not major concerns, many clients may not consistently receive optimal care [42]. This disconnect is now recognized as the ‘*know-do-gap*’ [43]. This aspect of quality improvement is more about ‘organizing processes of care’ within health facilities, and their catchment communities in order to optimize delivery of care. Some of the promising concepts to address these issues have been the deliberate application of improvement principles/approaches in healthcare settings [44]. At the time of advancement of the three-delay model, these concepts about quality improvement were largely untested in healthcare systems in LICS. Over the last decade, knowledge around the application of improvement approaches in healthcare has rapidly evolved. However, it is worth noting that most of the documented results have come from single-site facilities. Second, most of these have provided data on application of such principles to circumscribed services only (e.g increase facility delivery, or scale up HIV testing) not integrated health services that each facility in LICS needs to offer. Third, knowledge of the application of the said principles from a health systems strengthening perspective is very limited, making scale-up problematic [44].

Application of ‘improvement science’ in the health sector to drive ‘quality of care’ in LICS hardly featured as a core strategy and deliverable in the global health systems strengthening agenda during the time the three delay model was been advanced. Over the last two decades, although numerous demonstrations of how this can lead to improvement in service delivery have showed some success, evidence demonstrating large-scale application that can lead to national and global level impact is lacking as noted above [45–47]. Therefore, large scale implementation research is required to generate such knowledge. Such a research agenda should explore how to operationalize vertical scale up of ‘improvement science’ (methods for institutionalization at national and sub-national level), as well as horizontal scale up involving application of ‘improvement science’ at actual facility level (care micro-systems) to address quality gaps in integrated health services.

Largely, although the importance of ‘quality of care’ was recognized in the three-delay model, it has not elicited robust global attention. Furthermore, although the phrase ‘quality of care’ may feature in most health programs write up, very few articulate any strategy for achieving this at global, national or even care micro-systems (health facility) level [45–47]. It is likely that this may largely reflect lack of knowledge and skills on how to apply improvement methods to health care settings at programming level. Overall it is clear that ‘quality of care’ is central to achieving any global health goals. As noted above, it may therefore be necessary to invest in largescale operational research on continuous improvement of ‘quality of care’ in LICS, as a strategy in the post 2015 global health agenda and how it can be applied as a strategy to accelerate the progress towards ending preventable maternal and neonatal deaths.

### Prong 5: Post-discharge/hospitalization follow up and rehabilitation

Improved access and initial care may reduce early neonatal mortality but leave a sizeable proportion of infants who are more likely to succumb at a later stage [48]. It is likely that rudimentary post-discharge follow up and rehabilitation systems may contribute to a proportion of the post-discharge morbidity thus significantly hampering the overall impact of any investments to reduce maternal and more so neonatal mortality [48]. Of major concern too is that a sizeable proportion of neonates admitted and treated for various insults may be left with severe neuro-developmental impairments leading to severely constrained quality of life and requiring frequent re-admissions, further straining the meager health systems and family resources in LICS [48]. Evidence further suggests neonatal and perinatal insults maybe a major cause of behavioral and mental disorders among survivors which further require long-term care [48]. Overall without any focus on long-term rehabilitation and follow up, many of these children maybe dying prematurely during childhood, further impacting on the family and community, and adversely affecting overall life-expectancy and disability-adjusted life-years in LICS [48–51].

Besides the high post discharge mortality and long term neuro squeal from perinatal and neonatal insults, many women suffer long term complication especially during labor and delivery in low income countries such as obstetric fistulas among others [52, 53]. Estimates suggests that for every dead mother from pregnancy related complications in LICS, fifteen times more maybe left with long term grievous morbidity [52]. Despite this, programing based on the three delay model continue to largely focus on access and initial treatment with little systematic infrastructure for follow up and rehabilitation of women with such debilitating morbidity, with recent estimates indicating that nearly one million women are living with untreated obstetric fistulas globally [54].

Overall, despite the evidence that there is need to focus on post-discharge follow up, investments to prevent maternal and newborn morbidity and mortality in LICS have hardly focused on the post hospitalization period and rehabilitation. The prime focus has been skewed towards preventing ‘initial’ mortality. It is likely that in such a healthcare system where effort is limited to only preventing deaths at the initial client-facility contact, immediate neonatal care and outcomes may improve. However, this may be spurious, resulting from simply shifting a large share of the mortality burden to the post-discharge period and further creating an epidemic of severe impairment in fragile healthcare systems in LICS [48].

In summary, emerging evidence continues to suggest that even after preventing ‘initial’ mortality, a considerable number of neonates and young infants die after discharge from healthcare facilities in LICS [49]. Given that global, regional and in-country public health investments have predominantly applied the three-delay model in their programming, this aspect of the healthcare system in LICS is largely ignored. Consequently, post-hospitalization follow up and rehabilitation infrastructure (systems for follow up, human resource trained on rehabilitation post-sequelae) is largely non-existent [48]. Moving forward it is evident that continuing to ignore the need to strengthen the post discharge aspects of the healthcare systems in LICS will hamper any set goals, especially on reducing maternal and neonatal morbidity and mortality and improving the overall health and survival of children.

### Conclusion

The three-delay model has positively influenced global health-related development agenda, especially approaches towards achieving the prescribed aspirations of the now ending MDGs 4 & 5 over the last two decades. While it is recognized that many LICS have fallen short of the set targets within the MDGs time frame, appreciable gains have nevertheless been documented in reducing maternal and neonatal mortality. However, it apparent that, in moving to the post 2015 agenda, the scope of engagement in maternal and newborn health must be widened if these gains are to be sustained.

The five pronged approach we propose incorporates not only the need for continued investment in tackling the recognized three delays, but broadens this to also address earlier aspects of primary prevention, and the need for tertiary prevention through ongoing follow up and rehabilitation. Critically, it takes into perspective the spectrum of new evidence and how it can be deployed to deepen overall understanding of prevention strategies for maternal and neonatal morbidity and mortality in LICS. Certain aspects in all five prongs will require more investments in research to advance our understanding of fundamental aspects of maternal-neonatal morbidity and mortality, for example primary prevention of intrauterine hypoxemia and vaccine advances for diseases such as group B *Streptococcus* and HIV and AIDs. However, equally important is investment in the ‘science’ of how to consistently deliver the multitudes of known high impact interventions (health services research in LICS). The five pronged framework provides a ‘crystalline lens’ that can be used to aid the crafting and implementation of the post 2015 health-related development agenda especially in combating maternal and neonatal morbidity and mortality in LICS, and builds on the pillars of the three-delay model. The proposed framework brings public health approaches to improving maternal and new-born health in LICS closer to what is been practiced in the high middle, and high income regions in line with current evidence.

## Abbreviations

LICS: Low Income Countries
LMICS: Low and Middle Income countries
MMR: Maternal Mortality Ratio
MCH: Maternal and Child Health
MNCH: Maternal Newborn and Child Health
MDGs: Millennium Development Goals
HIV: Human Immunodeficiency Virus
AIDS: Acquired immunodeficiency Syndrome
LLITNs: Long lasting Insecticides Treated Mosquito Nets.
